# Physical properties of fish gelatin-based bio-nanocomposite films incorporated with ZnO nanorods

**DOI:** 10.1186/1556-276X-8-364

**Published:** 2013-08-27

**Authors:** Jalal Rouhi, Shahrom Mahmud, Nima Naderi, CH Raymond Ooi, Mohamad Rusop Mahmood

**Affiliations:** 1 Centre of Nanoscience and Nanotechnology (NANO-SciTech Centre), Institute of Science, Universiti Teknologi MARA, Shah Alam, Selangor 40450, Malaysia; 2 NANO-ElecTronic Centre, Faculty of Electrical Engineering, Universiti Teknologi MARA, Shah Alam, Selangor 40450, Malaysia; 3 Nano-Optoelectronic Research (NOR) Lab, School of Physics, Universiti Sains Malaysia, Pulau, Pinang 11800, Malaysia; 4 Department of Physics, University of Malaya, Kuala Lumpur 50603, Malaysia

**Keywords:** ZnO nanorods, Fish gelatin bio-nanocomposite films, UV shielding

## Abstract

Well-dispersed fish gelatin-based nanocomposites were prepared by adding ZnO nanorods (NRs) as fillers to aqueous gelatin. The effects of ZnO NR fillers on the mechanical, optical, and electrical properties of fish gelatin bio-nanocomposite films were investigated. Results showed an increase in Young's modulus and tensile strength of 42% and 25% for nanocomposites incorporated with 5% ZnO NRs, respectively, compared with unfilled gelatin-based films. UV transmission decreased to zero with the addition of a small amount of ZnO NRs in the biopolymer matrix. X-ray diffraction showed an increase in the intensity of the crystal facets of (10^ī^1) and (0002) with the addition of ZnO NRs in the biocomposite matrix. The surface topography of the fish gelatin films indicated an increase in surface roughness with increasing ZnO NR concentrations. The conductivity of the films also significantly increased with the addition of ZnO NRs. These results indicated that bio-nanocomposites based on ZnO NRs had great potentials for applications in packaging technology, food preservation, and UV-shielding systems.

## Background

The combination of nanostructures and biomaterials provide an unrivaled opportunity for researchers to find new nanobiotechnology areas. Nanorods (NRs) and nanoparticles combined with biomolecules are used for various applications in biomolecular sensors [1], bioactuators [2], and medicines, such as in photodynamic anticancer therapy [3].

Metal oxides, such as ZnO, MgO, and TiO_2_, are used extensively to construct functional coatings and bio-nanocomposites because of their stability under harsh processing conditions and safety in animal and human applications [4]. Moreover, these materials offer antimicrobial, antifungal, antistatic, and UV-blocking properties [5]. TiO_2_/Ag, ZnO-starch, and ZnO/SiO_2_/polyester hybrid composites have been investigated for UV-shielding textile coatings. TiO_2_ is more efficient in photoactivity when TiO_2_ precursor coatings are heat treated at 400°C [6]. However, such a process complicates the production of TiO_2_ UV-active coatings for textiles. ZnO has better advantages than TiO_2_ because ZnO can block UV in all ranges (UV-A, UV-B, and UV-C). Furthermore, functional nano-ZnO displays antibacterial properties in neutral pH even with small amounts of ZnO. ZnO nanostructures can be simply grown by chemical techniques under moderate synthesis conditions with inexpensive precursors. ZnO nanostructures in various morphologies, such as discs, rods, tubes, spheres, and wires, have been easily synthesized by the precipitation of surfactants followed by hydrothermal processes (120°C) and low temperature thermolysis (80°C) [7,8].

The use of gelatin as an organic additive in composites with inorganic nanohybrids has recently gained increasing interest because of the bioadhesive and biodegradable properties of gelatin [9].

Thus, several experts have concentrated their research on gelatin films made from mammalian sources, such as porcine and bovine. Mammalian gelatin films commonly have excellent mechanical properties compared with other types of gelatin films. Current researchers have focused on the use of marine gelatin sources as alternatives to mammalian gelatins, such as those from fish. Marine gelatin sources are not related to the risk of bovine spongiform encephalopathy. Furthermore, fish gelatin can be used with minimal religious prohibition in Islam, Judaism, and Hinduism [10].

In this paper, ZnO NRs were used as fillers to prepare fish gelatin bio-nanocomposites. The films were characterized for their mechanical, electrical, and UV absorption properties.

## Methods

### Materials

A total of 240 bloom fish gelatin was supplied by Sigma Chemical Co. (St. Louis, MO, USA). Glycerol and liquid sorbitol were purchased from CIM Company Sdn. Bhd. (Ipoh, Perak Darul Ridzuan, Malaysia).

### Synthesis of ZnO NRs

ZnO NRs were produced in a modification process known as the catalyst-free combust-oxidized mesh (CFCOM) process, which involves capturing the suboxide of zinc (ZnO_x_) at 940°C to 1,500°C followed by an air-quenching phase. The CFCOM process was performed using a factory furnace.

**Figure 1:**
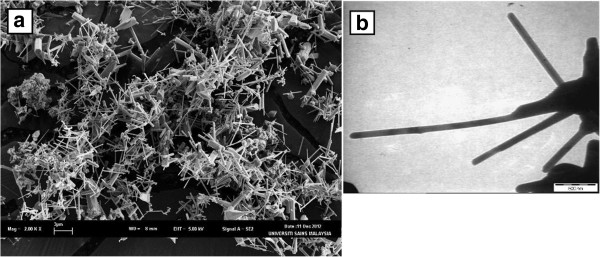
FESEM (a) and TEM (b) images of ZnO nanorods synthesis by CFCOM process.

The field-emission scanning electron microscopy micrographs in Figure 1 show that the high surface area ZnO powder is composed of rod-like clusters. In our previous work [11,12], we found that hexagonal rods are the preferred morphological configuration in localized areas that are comparatively rich in oxygen content, whereas rectangular nanoplates/boxes are preferred in localized regions with comparatively low oxygen partial pressures.

ZnO NRs were observed in different lengths and widths because of the large variety in growth conditions in the CFCOM process. Figure 1b illustrates the transmission electron microscopy micrographs of ZnO NR clusters with 0.5 to 2 μm lengths and 50 to 100 nm diameters.

### Preparation of ZnO bio-nanocomposite films

ZnO NRs were added to distilled water at different concentrations. The mixture was heated at 70°C ± 5°C for approximately 45 min with constant stirring to dissolve the ZnO NRs completely. Thereafter, the mixture was exposed in an ultrasonic bath for 20 min. The solution was cooled to ambient temperature and was used to prepare 5 wt.% aqueous gelatin. Sorbitol (0.15 *g*/*g* gelatin) and glycerol (0.15 *g*/*g* gelatin) were added as plasticizers. The gelatin nanocomposites were heated to 55°C ± 5°C and held for 45 min. The gelatin nanocomposite solution was then cooled to 40°C, and the bubbles were removed using a vacuum.

A portion (90 g gelatin) of the dispersion was cast onto Perspex plates (England, UK) (150 mm × 150 mm × 3 mm). The composite films of the gelatin/ZnO NRs were dried at 50% ± 5% relative humidity (RH) and 24°C ± 1°C for 24 h. Control films were prepared with the same plasticizers but without nanostructures. Dried films were manually removed and conditioned at approximately 25°C ± 1°C and 52% ± 2% RH in a desiccator for further analysis. All films (including control) were prepared in triplicate.

### Characterization

The mechanical properties of the bio-nanocomposite films (such as tensile strength (TS), elongation at break (EAB), and Young's modulus (YM)) and the seal strength of the heat-sealed films were determined using a texture analyzer equipped with Texture Exponent 32 V.4.0.5.0 (TA.XT2, Stable Micro System, Godalming, Surrey, UK) according to ASTM D882-10 (American Society for Testing and Materials, 2010). The initial grip length and crosshead speed were 50 mm and 0.5 mm/s, respectively. EAB and TS at break were calculated from the deformation and force data recorded by the software.

The UV-vis spectra of the gelatin/ZnO NR bio-nanocomposite films were recorded using a UV-vis spectrophotometer (UV-1800, Shimadzu, Kyoto, Japan). A high-resolution X-ray diffraction (XRD) system (X'Pert PRO Materials Research Diffractometer PW3040, PANalytical, Almelo, The Netherlands) was used to investigate the crystalline structures. A Fourier transform infrared (FTIR) spectrometer (Spectrum GX FTIR, Perkin Elmer, Waltham, MA, USA) was used in this study for absorption spectroscopy. The conductivity properties of fish gelatin-based nanocomposites were examined using an Agilent 4284a Precision LCR meter (Santa Clara, CA, USA) in the frequency range of 0.01 and 1,000 kHz.

The surface topography of the films was measured by atomic force microscopy (AFM) (Dimension Edge, Bruker, Madison, WI, USA) with a contact operation mode. The surface roughness of the films was calculated based on the root mean square deviation from the average height of the peaks after subtracting the background using Nanoscript software (Veeco Instruments, Plainview, NY, USA) according to ASME B46.1.14.

## Results and discussion

**Figure 2:**
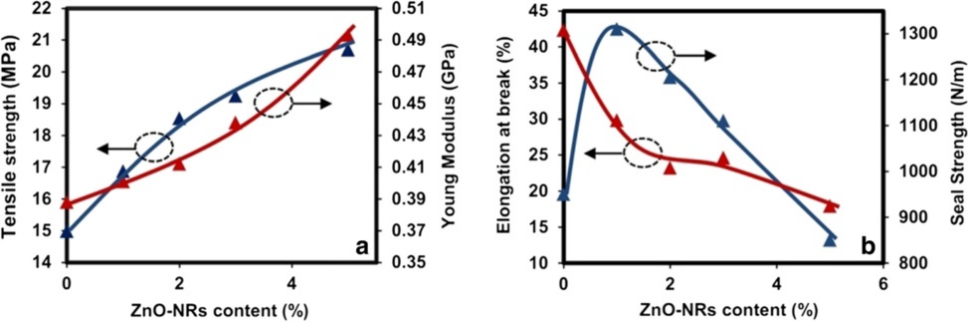
**Effects of ZnO NR contents on the mechanical properties of gelatin nanocomposite films.** Effects of ZnO NR contents on **(a)** tensile strength and Young's modulus and **(b)** elongation at break and seal strength of gelatin nanocomposite films.

Figure 2a shows the TS and YM. A significant increase in both TS and YM was observed and was consistent with other studies on reinforced biopolymer film by nanoparticles [13]. EAB decreased with the addition of ZnO NRs (Figure 2b), which could be attributed to the moisture content and interfacial interaction between the ZnO NRs and biopolymer matrix. Water plays a plasticizing role in biocomposite films. By contrast, decreasing the plasticizer content increases TS and YM and decreases EAB [14]. The mechanical properties of the biopolymer matrix have been reported to be extremely dependent on the interfacial interaction between the fillers and the matrix [15].

Although heat sealability is an important factor for packaging materials, only a few studies have investigated this topic. The seal strength for gelatin matrices increased with lower concentrations of ZnO NRs (Figure 2b). This result was attributed to the improvement of hydrogen and other bonds on the ZnO NR surface. However, the sealability of the films decreased with addition of higher percentage of ZnO NRs, possibly due to the reduction in flexibility and moisture content of the films.

**Figure 3:**
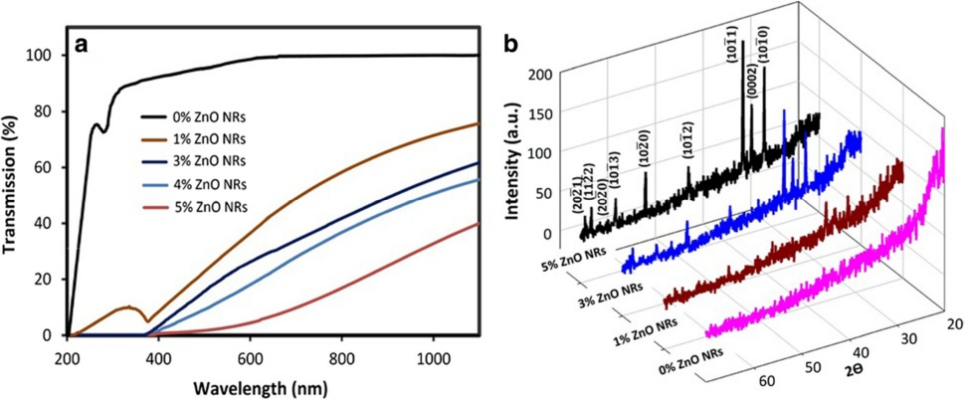
**UV-vis transmission spectra and X-ray diffraction of fish gelatin-based bio-nanocomposite films. (a)** UV-vis spectra at the wavelength range of 200 to 1,100 nm of the gelatin films with ZnO NRs at various concentrations. **(b)** XRD patterns for the gelatin nanocomposite films with various concentrations of ZnO NRs.

The UV-vis spectra at the wavelength range of 200 to 1,100 nm of the gelatin films with ZnO NRs at various concentrations are shown in Figure 3a. The control films showed very high transmittance in the UV range of 290 to 400 nm. UV transmission decreased (almost 0%) with the addition of a very low amount of ZnO NRs to the biopolymer matrix, thus indicating that the films incorporated with ZnO NRs had lower transmission in the UV range.

Yu et al. [16] reported that the biocomposite films incorporated with 5% ZnO nanoparticles increased the UV light absorption unit to 2.2, whereas the UV at the same level was absorbed with the addition of low amounts of ZnO NRs. The different behavior of ZnO NRs in the present study could be attributed to the shape and crystal structure of ZnO NRs. The XRD patterns for the gelatin nanocomposite films with various concentrations of ZnO NRs are shown in Figure 3b. In higher ZnO NRs concentrations, the major XRD diffraction peaks of (10^ī^0), (0002), and (10^ī^1) appeared strong and narrow, thus suggesting the existence of a high-level ZnO crystalline structure.

The UV adsorption rate of the biocomposite films can also be related to the intensity of the crystal facets of (10^ī^1) and (0002) (Figure 3b). These crystal facets are highly excitonic at the UV near band edge regime [12], thus indicating that a biopolymer matrix incorporated with ZnO NRs could be used as heat insulator and UV-shielding film in the packaging industry.

**Figure 4:**
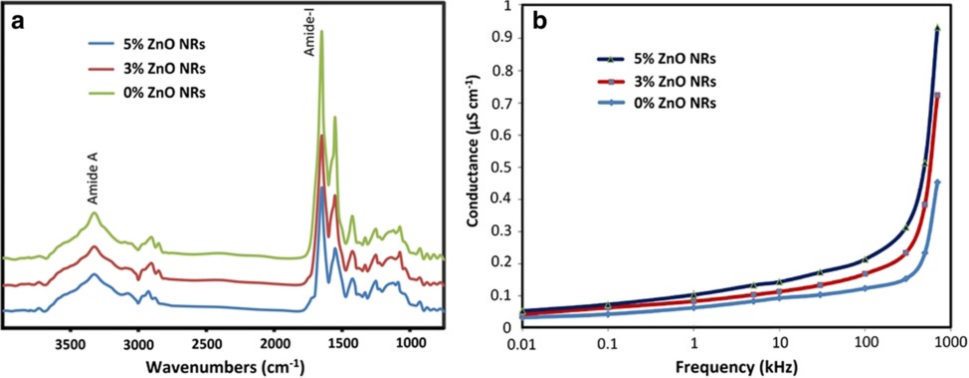
**FTIR absorption spectra and conductivity of fish gelatin-based bio-nanocomposite films filled with ZnO NRs. (a)** FTIR spectra of the gelatin films incorporated with ZnO NRs at selected concentrations. **(b)** Conductivity variations with frequencies at various concentrations of ZnO NR-incorporated fish gelatin films.

The FTIR spectra of the gelatin films incorporated with ZnO NRs at selected concentrations are shown in Figure 4a. The obtained peaks were related to the amide band regions, which were contributed by the gelatin. All biocomposite films had major peaks in the amide region, which presented small differences in the spectra. The control film, 3% ZnO NRs, and 5% NR-incorporated fish gelatin films exhibited the amide-I bands at the wavenumbers of 1,648.78, 1,644.56, and 1,644.35 cm^−1^, respectively.

The FTIR spectra differences between various samples in the amide-I region were mainly relatesd to the different orientations and conformations of the polypeptide chains affected by the incorporation of ZnO NRs. The shifts of the amide-I peak to a lower wavenumber were related to a decrease in the molecular order because of conformational change. Furthermore, the amide-A band from the N-H stretching vibration of the hydrogen-bonded N-H group became visible at wavenumbers 3,298.78, 3,297.25, and 3,295.89 cm^−1^ for the control film, 3% ZnO NRs, and 5% ZnO NR-incorporated fish gelatin films, respectively. The position of the band in the amide-A region shifts to lower frequencies when N-H groups with shorter peptides are involved in hydrogen bonding [17]. In the present research, the amide-A band shifted to lower frequencies when the ZnO NR concentration increased from 0% to 5%. This result clearly showed that the N-H groups from shorter peptide fragments produced hydrogen bonding within the fish gelatin films.

Figure 4b shows the conductivity variations with frequencies at various concentrations of ZnO NR-incorporated fish gelatin films. The conductivity of the control films was less than the gelatin films filled with ZnO NRs. Furthermore, the conductivity significantly increased with increasing filler concentration. The conductivity displays a dispersion frequency independent behavior at higher and low frequency regions. The maximum conductivity of 0.92 × 10^−6^ S cm^−1^ was observed for fish gelatin films incorporated with 5% ZnO NRs.

Certain factors may influence conductivity, including the mobility of free charges, number of charge carriers, and availability of connecting polar domains as conduction pathways [18]. In bio-nanocomposite films, the increase in conductivity values can be attributed to the increase in charge carriers because of the incorporation of ZnO NRs in the biocomposite matrices.

**Figure 5:**
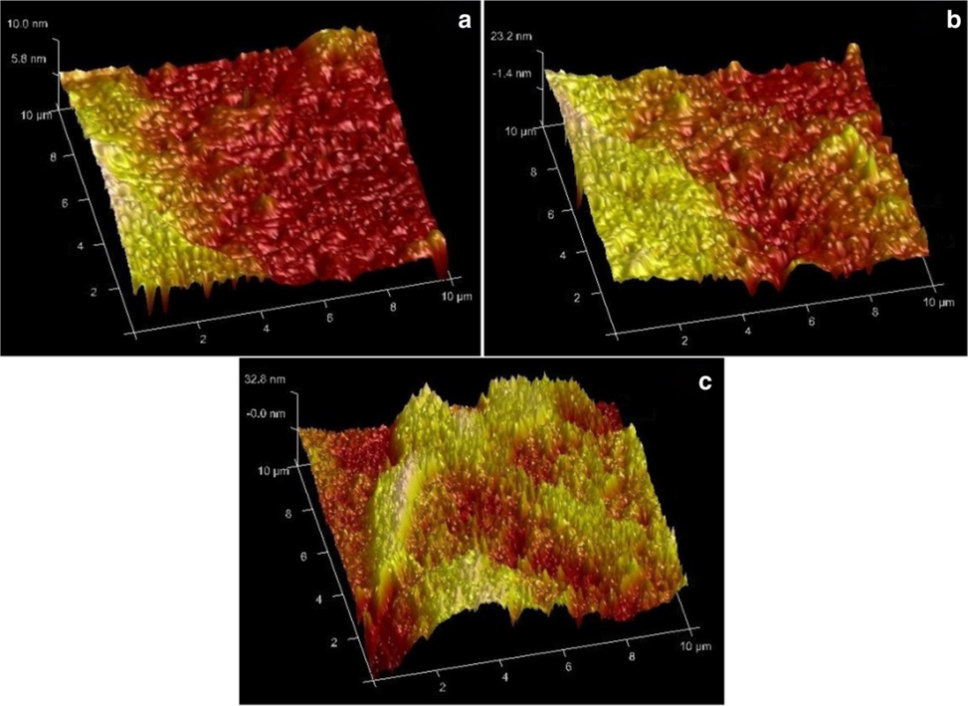
**AFM surface morphology of fish gelatin films.** AFM surface morphology of fish gelatin films for the **(a)** control film, **(b)** 3% ZnO NRs, and **(c)** 5% ZnO NRs incorporated.

Based on the AFM analysis corresponding to the three samples (Figure 5), the average roughness height were 56.8, 94.3, and 116.7 nm for the control film, 3% ZnO NRs, and 5% ZnO NRs, respectively. The increase in surface roughness with increasing ZnO NR concentration could be attributed to the physical interaction between ZnO NRs and fish gelatin. No new functional group appeared after the application of ZnO NRs (Figure 4a), thus indicating that only physical interaction occurred between the ZnO NRs and the film matrix.

## Conclusions

ZnO NRs played an important role in enhancing the physical properties of fish gelatin-based biocomposites. After the incorporation of low levels of ZnO NR fillers, significant differences were observed in the film properties, particularly in electrical, mechanical, and UV protection activities. The optical properties of bio-nanocomposites indicated that the UV transmission becomes almost zero with the addition of small amounts of ZnO NRs to the biopolymer matrix. The presence of ZnO NRs in fish gelatin-based polymers enabled the localization of charge carriers, thus improving the electrical properties of conventional polymers. The FTIR spectra indicated the physical interaction between the gelatin and ZnO NRs. XRD diffraction shows that the intensity of the crystal facets of (10^ī^1) and (0002) increased with increasing ZnO NR concentrations in the biocomposite matrix. These crystal facets also increased the UV absorption. Therefore, ZnO biopolymer nanocomposites have excellent potential applications in food packaging and UV shielding.

## Abbreviations

AFM: Atomic force microscopy; CFCOM: Catalyst-free combust-oxidized mesh; EAB: Elongation at break; FTIR: Fourier transform infrared; NR: Nanorod; TS: Tensile strength; XRD: X-ray diffraction; YM: Young’s modulus.

## Competing interests

The authors declare that they have no competing interests.

## Authors’ contributions

JR carried out the experimental work and characterizations of the sample, analyzed all the data, and wrote the manuscript. SM and NN participated in the experimental work, characterization, and coordination. CHRO improved the manuscript and participated in the studies. MRM supervised the research work. All authors read and approved the final manuscript.
